# Design and simulation of a graphene-integrated SPR biosensor for malaria detection

**DOI:** 10.3389/fbioe.2025.1580344

**Published:** 2025-06-23

**Authors:** Talia Tene, Fabian Arias Arias, Karina I. Paredes-Páliz, Juan Carlos González García, Nataly Bonilla García, Cristian Vacacela Gomez

**Affiliations:** ^1^ Department of Chemistry, Universidad Técnica Particular de Loja, Loja, Ecuador; ^2^ Department of Chemistry and Chemical Technologies, University of Calabria, Arcavacata, Italy; ^3^ Grupo de Investigación en Salud Pública, Facultad de Ciencias de la Salud, Universidad Nacional de Chimborazo, Riobamba, Ecuador; ^4^ Facultad de Ciencias, Escuela Superior Politécnica de Chimborazo (ESPOCH), Riobamba, Ecuador; ^5^ INFN-Laboratori Nazionali di Frascati, Frascati, Italy

**Keywords:** surface plasmon resonance, kretschmann configuration, transfer matrix method, silicon nitride, graphene, biosensors, malaria

## Abstract

This work presents the theoretical design and optimization of a surface plasmon resonance (SPR) biosensor incorporating graphene, silicon nitride, and a thiol-tethered ssDNA layer for malaria detection and stage differentiation. Two configurations (Sys_3_ and Sys_4_) were simulated using the transfer matrix method to determine optimal material thicknesses. The final designs were evaluated against three malaria stages—ring, trophozoite, and schizont—based on their refractive index variations. Sys_3_ achieved sensitivities of 353.14, 291.14, and 263.26°/RIU, while Sys_4_ reached 315.71, 294.81, and 268.65°/RIU, respectively. These values exceed those reported in comparable SPR platforms. Sys_3_ showed enhanced optical performance with a higher quality factor and lower detection limit, whereas Sys_4_ offered improved biomolecular recognition. Although limited to simulation, the proposed configurations demonstrate potential for label-free, stage-specific malaria diagnostics, supporting future development toward point-of-care applications.

## 1 Introduction

Malaria remains a major public health concern in tropical and subtropical regions, with Plasmodium falciparum causing the most severe clinical manifestations (Kogan, 2020). The disease progresses through intraerythrocytic stages (ring, trophozoite, and schizont), each marked by distinct biochemical and morphological changes in infected red blood cells (RBCs) (Bendib and Bendib, 2018; Agnero et al., 2019). These changes alter the refractive index (RI) of the RBCs (Pravesh et al., 2024), which can be exploited for label-free optical detection. Timely and accurate diagnosis is critical for reducing morbidity, limiting transmission, and guiding effective treatment.

Current malaria diagnostics, including light microscopy (Payne, 1988), rapid diagnostic tests (RDTs) (Mukkala et al., 2018), and nucleic acid amplification methods (Proux et al., 2011), each present limitations. Light microscopy remains the gold standard due to species differentiation and parasitemia quantification (Abbas et al., 2018), but it is time-consuming and operator-dependent. RDTs target specific antigens such as HRP2 and LDH (Chakma et al., 2023), yet their reliability may be compromised by gene deletions and residual antigenemia. Molecular approaches like PCR (Fallon et al., 2003) and LAMP (Morris and Aydin-Schmidt, 2021) offer improved sensitivity and specificity but require skilled personnel and access to specialized equipment, increasing cost and limiting field applicability.

Surface plasmon resonance (SPR) biosensors offer an attractive alternative for malaria detection (Kumar et al., 2024). These sensors utilize surface plasmon waves, which are collective oscillations of conduction electrons at a metal–dielectric interface (Philip and Kumar, 2022; Stefancu et al., 2024), to monitor refractive index changes near the sensor surface. The Kretschmann configuration remains the most widely adopted SPR geometry due to its simplicity and effective excitation of surface plasmons (Shukla et al., 2022). By tracking shifts in the resonance angle, SPR biosensors enable real-time, label-free detection of malaria biomarkers (Wang et al., 2022). They are also amenable to miniaturization for point-of-care (POC) applications (Rasheed et al., 2024).

Traditional SPR sensors often employ noble metals such as gold (Au) or silver (Ag) as the plasmonic layer (Lee and El-Sayed, 2006). Although Au offers superior chemical stability, Ag provides sharper resonance peaks and stronger plasmonic fields (Naik et al., 2013), making it advantageous for improving sensor resolution when chemical degradation is not a primary concern.

To overcome intrinsic losses in metallic layers and enhance biomolecular interaction, recent studies have incorporated two-dimensional (2D) materials, especially graphene, into SPR platforms (Tene et al., 2024a). Graphene’s high surface area, exceptional conductivity, and tunable optical properties support improved field confinement and molecular adsorption (Coello-Fiallos et al., 2017). Moreover, the addition of a dielectric interlayer such as silicon nitride (Si_3_N_4_), which has a high refractive index and low optical loss, can increase electromagnetic confinement and structural stability (Tene et al., 2024b).

The functionalization of graphene with single-stranded DNA (ssDNA) enables specific biorecognition of malaria DNA sequences (Ragav et al., 2018). Thiol-tethered ssDNA, in particular, facilitates stable and oriented binding to sensor surfaces and promotes efficient hybridization with target sequences (Wang et al., 2004). Compared to antibody-based strategies, ssDNA-functionalized interfaces demonstrate enhanced stability, reproducibility, and resistance to environmental degradation.

Malaria stage differentiation is also feasible using SPR biosensors due to the distinct RI signatures of RBCs at various infection stages. The ring stage (II) exhibits a moderate RI decrease due to vacuole formation and low metabolic activity (Kozicki et al., 2015). In the trophozoite stage (III), hemoglobin digestion and hemozoin production reduce the RI further (Anderson et al., 2015). The schizont stage (IV), marked by parasite replication and RBC rupture, displays the lowest RI due to extensive structural degradation (Roobsoong et al., 2011). These progressive changes can be detected by monitoring resonance angle shifts in the SPR response.

This study presents the theoretical design and simulation of a graphene-integrated SPR biosensor configured with a multilayer structure comprising silver, silicon nitride, graphene, and thiol-tethered ssDNA. Particularly, we complement and extend our previous work, which uses as the sensing system: BK7/Silver/Silicon Nitride/Black Phosphorus (Tene et al., 2025a). Hence, the sensor response is modeled using the transfer matrix method (TMM), a well-established approach for analyzing electromagnetic behavior in multilayer thin films (Tene et al., 2024c). Key performance parameters, including sensitivity (S), full-width at half maximum (FWHM), quality factor (QF), detection accuracy (DA), figure of merit (FoM), limit of detection (LoD), and comprehensive sensitivity factor (CSF), are evaluated. The sensor’s capability to resolve malaria stages (ring, trophozoite, schizont) is assessed based on RI variation, offering a theoretical basis for future label-free, stage-specific malaria diagnostics.

## 2 Materials and methods

### 2.1 Theoretical framework

The reflective intensity of the proposed *N* ^*th*^-layer sensor model is calculated using the TMM (Wu et al., 2010; Tene et al., 2024d; Akib et al., 2024). The analysis of the sensor considers boundary conditions for the tangential component, with initial limit Z = Z_1_ = 0, and final limit Z_n-1_, giving the following expression:

$$\left[\begin{array}{c} E_{1}\\ H_{1} \end{array}\right]=M_{ij}\left[\begin{array}{c} E_{N-1}\\ H_{N-1} \end{array}\right]$$ (1)

In Equation 1, *E*_*1*_, *E*_*N-1*_, *V*_*1*_, and *V*_*N-1*_ represent the tangential components of the electric and magnetic fields for the initial and *Nth* layer, respectively. M_*ij*_ indicates the transfer matrix characteristics of the *Nth* layer model. The transfer matrix can be computed as:

$$M_{ij}={\left[\prod_{k=2}^{N-1}M_{k}\right]}_{ij}=\left[\begin{array}{cc} M_{11} & M_{12}\\ M_{21} & M_{22} \end{array}\right]$$ (2)

To be specific, the matrix *M*_*k*_ (Equation 2) is the characteristic transfer matrix for the *k*th layer in a multilayer thin-film structure. It relates the forward and backward traveling electromagnetic field components at the entry and exit of that specific layer, as follows:

$$M_{k}=\left[\begin{array}{cc} \cos\beta_{k} & \left(-i\;\sin\beta_{k}\right)/q_{k}\\ -i\;q_{k}\;\sin\beta_{k} & \cos\beta_{k} \end{array}\right]$$ (3)

Denoting the phase thickness, $\beta_{k}$ , as:

$$\beta_{k}=\frac{2\pi d_{k}}{\lambda_{0}}\sqrt{\varepsilon_{k}-n_{1}^{2}\sin^{2}\theta }$$ (4)

And the polarization-dependent wave impedance factor, $q_{k}$ , is denoted as:

$$q_{k}=\frac{\sqrt{\varepsilon_{k}-n_{1}^{2}\sin^{2}\theta }}{\varepsilon_{k}}$$ (5)

in Equations 3–5, $\lambda_{0}$ represents the wavelength of the incident light, $n_{1}$ is the refractive index, $\varepsilon_{k}$ represents the dielectric constant, $\beta_{k}$ represents the phase constant, θ represents the entrance angle, and $d_{k}$ represents the depth of the $k^{th}$ layer. For comparison with experiments, we adopt the use of He-Ne laser with $\lambda_{0}=633$ nm.

After straightforward computations, the total reflection of the *N* ^*th*^-layer model can be expressed as:

$$R={\left|\frac{\left(M_{11}+M_{12}\;q_{N}\right)q_{1}-\left(M_{21}+M_{22}\;q_{N}\right)}{\left(M_{11}+M_{12}\;q_{N}\right)q_{1}+\left(M_{21}+M_{22}\;q_{N}\right)}\right|}^{2}$$ (6)

By using Equation 6, the reflectance as a function of the angle of incidence (SPR curve) can be calculated as the main ingredient fo the current research.

### 2.2 Performance metrics equations

We now move on the main performance metric (Equations 7–13) of the proposed sensors (Tene et al., 2025b). The first parameter is the sensitivity enhancement regarding the “relative” baseline sensors after/before pathogen adsorption, denoted as:

$$∆S_{RI}^{after}=\frac{\left(S_{RI}^{after}-S_{RI}^{before}\right)}{S_{RI}^{before}}$$ (7)

the sensitivity to the refractive index change can be expressed as:

$$S_{RI}=\frac{∆\theta }{∆n}$$ (8)

Here, ∆θ represents the angle shift variation and ∆n represents the refractive index variation.

The detection accuracy (DA) can be expressed as in terms of ∆θ and the full width at half maximum (FWHM) of the SPR curve, as:

$$DA=\frac{∆\theta }{FWHM}$$ (9)

The Quality Factor (QF) can be expressed in terms of $S_{RI}$ and FWHM, as follows:

$$QF=\frac{S_{RI}}{FWHM}$$ (10)

The Figure of Merit (FoM) can be expressed as:

$$FoM=\frac{S_{RI}\left(1-R_{\min}\right)}{FWHM}$$ (11)

Here, $R_{\min}$ represents the lowest normalized reflection value of the SPR curve.

The Limit of Detection (LoD) can be calculated as:

$$LoD=\frac{∆n}{∆\theta }\times 0.005^{{}^{\circ}}$$ (12)

In Equation 12, the value 0.005° represents the angular resolution of typical SPR systems. This value reflects the minimum detectable angle shift in conventional setups and is commonly used in theoretical models as a conservative estimate of instrumental precision.

Finally, the Comprehensive Sensitivity Factor (CSF) ratio can be calculated:

$$CSF=\frac{S_{RI}\times \left(R_{\max}-R_{\min}\right)}{FWHM}$$ (13)

$R_{\min}$ represents the maximum reflectance before resonance, typically at non-resonant wavelengths or angles. All computations in this investigation are done with a data sampling of $5\times 10^{4}$ points.

### 2.3 Biosensor design

Table 1 outlines the five SPR biosensor configurations considered in this study, systematically incorporating functional layers to evaluate their influence on the sensor performance. The baseline system, Sys_0_ (P/Ag/M_Blood_), consists of a prism-silver-plasma blood structure, serving as a reference for assessing SPR behavior in a biological medium (Karki et al., 2024). In Sys_1_ (P/Ag/Stage_1_), the sensing medium is replaced with normal (I) stage erythrocytes, providing a biologically relevant model for investigating refractive index variations associated with malaria (Bendib and Bendib, 2018; Agnero et al., 2019). The addition of Si_3_N_4_ in Sys_2_ (P/Ag/SN/Stage_1_) is expected to enhance the plasmonic confinement, while graphene in Sys_3_ (P/Ag/SN/G/Stage_1_) is expected to improve the biomolecular interactions. The most advanced system, Sys_4_ (P/Ag/SN/G/ssDNA/Stage_1_), integrates a thiol-tethered ssDNA layer, which is expected to selectively bind *Plasmodium* DNA sequences.

**TABLE 1:** Configurations of the SPR biosensors evaluated in this study, using different materials and sensing media. The “Full Name” column describes the structure from bottom to top.

Sys No.	Code	Full name	Nick name
0	Sys_0_	Prism/Silver/Plasma Blood	P/Ag/M_Blood_
1	Sys_1_	Prism/Silver/Normal (I)	P/Ag/Stage_I_
2	Sys_2_	Prism/Silver/Si_3_N_4_/Normal (I)	P/Ag/SN/Stage_I_
3	Sys_3_	Prism/Silver/Si_3_N_4_/Graphene/Normal (I)	P/Ag/SN/G/Stage_I_
4	Sys_4_	Prism/Silver/Si_3_N_4_/Graphene/ssDNA/Normal (I)	P/Ag/SN/G/ssDNA/Stage_I_

**FIGURE 1:**
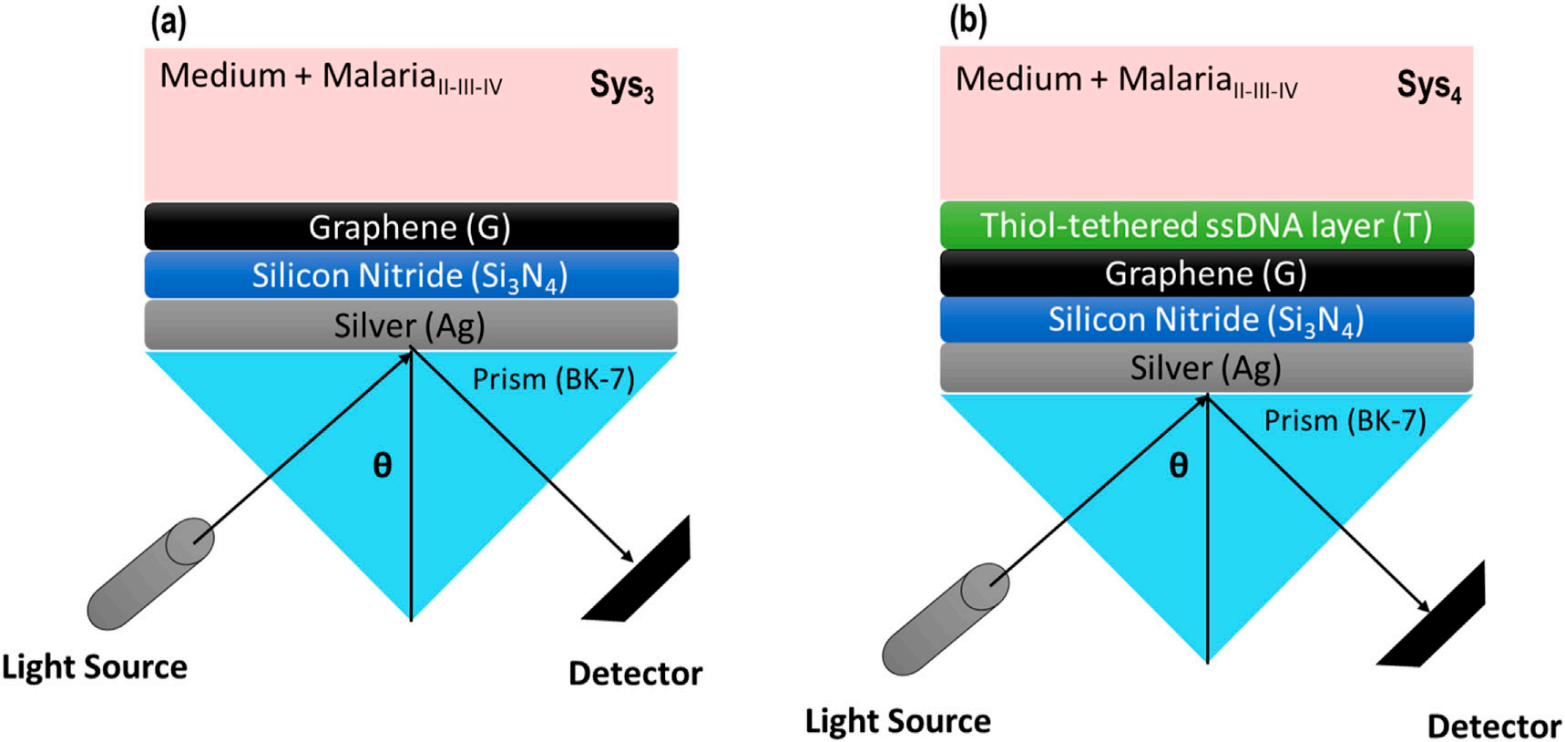
Schematic representation of the proposed SPR biosensor configurations. **(a)** Configuration with silicon nitride and graphene layers. **(b)** Configuration with silicon nitride and graphene layers, incorporating a thiol-tethered ssDNA layer for enhanced biorecognition.

To note, Figure 1 provides a schematic representation of the advanced SPR biosensor configurations, Sys_3_ and Sys_4_, highlighting their layered structure and optical setup. In Sys_3_ (Figure 1a), the system consists of a silver layer deposited on a BK-7 prism, followed by a silicon nitride layer and a graphene monolayer, with the analyte medium positioned at the top. The Kretschmann configuration is employed, where light is incident at an angle θ, exciting surface plasmons at the silver-dielectric interface. The inclusion of Si_3_N_4_ and graphene is expected to improve resonance sharpness and enhance molecular interaction sensitivity. In Sys_4_ (Figure 1b), the configuration is extended by incorporating a thiol-tethered ssDNA functionalisation layer atop the graphene surface. The structural progression from Sys_0_ to Sys_4_ follows a logical design approach, ensuring that each modification contributes to enhanced sensor performance.

### 2.4 Initial parameters

Table 2 presents the initial refractive index (RI) and thickness values adopted for the SPR biosensor configurations before optimisation. These values have been taken from experimental and theoretical studies reported in the literature at around 633 nm. The materials used in the sensor’s structure include BK-7 glass (prism), silver (Ag), silicon nitride (Si_3_N_4_), graphene (G), plasma blood, and normal (I) stage erythrocytes. The BK-7 prism, which serves as the coupling medium, has a refractive index of 1.5151, a standard value for optical applications due to its high transparency in the visible and near-infrared spectrum. The silver layer, acting as the plasmonic medium, exhibits a complex refractive index of 0.056253 + 4.2760*i* at the operating wavelength, with a thickness of 55.0 nm, ensuring optimal plasmon resonance excitation. The Si_3_N_4_ dielectric layer has a refractive index of 2.0394 and a thickness of 5.00 nm, parameters selected based on its optical properties and integration compatibility with plasmonic 2D nanomaterials. The graphene monolayer is characterised by a refractive index of 3.0 + 1.462*i*, with a thickness of 0.34 nm (Wu et al., 2010), consistent with its atomic-layer nature.

**TABLE 2:** Initial parameters adopted in the SPR biosensor configuration before optimization. The refractive index (RI) and thickness values for each material used in the sensor’s construction are shown at 633 nm.

Material	Refractive index	Thickness (nm)	Ref.
BK-7 (P)	1.5151	---	Tene et al. (2024b)
Silver (Ag)	0.056253 + 4.2760 *i*	55.0	Tene et al. (2024c)
Si_3_N_4_ (SiN)	2.0394	5.00	Kumar et al. (2022)
Graphene (G)	3.0 + 1.462 *i*	0.34	Wu et al. (2010)
Plasma blood	1.340	---	Karki et al. (2024)
Normal (I) stage (erythrocytes)	1.402	---	Bendib and Bendib (2018), Agnero et al. (2019)

The sensing media, i.e., plasma blood and normal (I) stage erythrocytes, exhibit refractive indices of 1.340 and 1.402, respectively, reflecting their inherent optical differences. These values are essential for evaluating the biosensor’s response across different biological conditions and Malaria stages.

To clarify, this study evaluates the biosensor response based on discrete refractive index values associated with the ring, trophozoite, and schizont stages of *Plasmodium falciparum* infection, as reported in prior works (Bendib and Bendib, 2018; Agnero et al., 2019). These values do not account for concentration-dependent behavior typically represented by sigmoidal dose–response curves. As such, output versus analyte concentration was not modeled. A full biochemical analysis incorporating binding kinetics and concentration gradients remains outside the scope of this theoretical framework and is proposed for future experimental validation.

## 3 Results and discussions

### 3.1 Configurations under analysis

**FIGURE 2:**
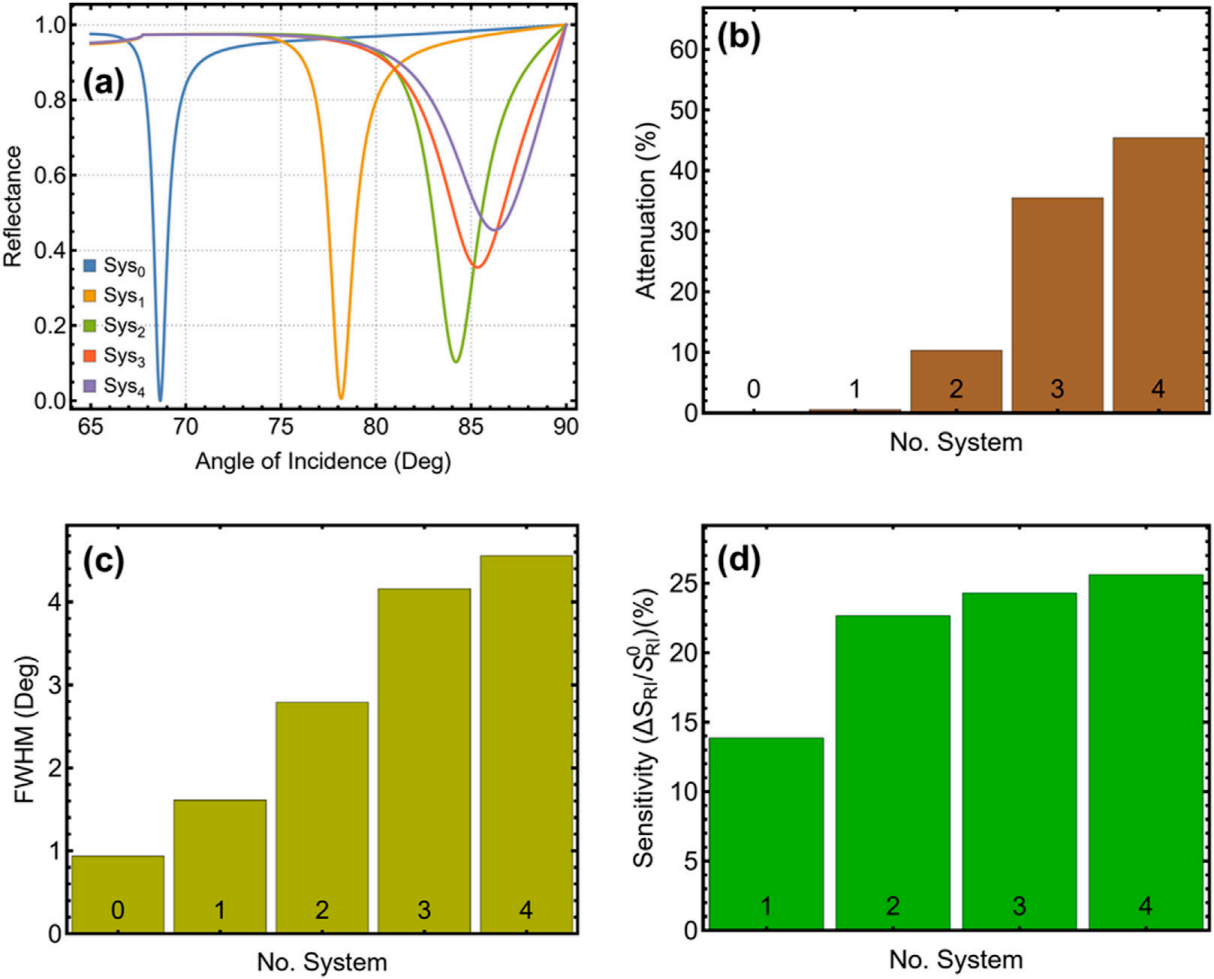
Performance analysis of the SPR biosensor configurations. **(a)** SPR reflectance curves for different configurations from Sys_0_ to Sys_4_. **(b)** Percentage of attenuation for different configurations. **(c)** FWHM for different configurations. **(d)** Sensitivity enhancement (%) for configurations Sys_1_-Sys_4_ compared to the reference configuration Sys_0_.

Figure 2 and Supplementary Table S1 present the graphical and numerical evaluation of the different SPR biosensor configurations, illustrating the impact of material modifications on SPR peak position, attenuation, FWHM, and sensitivity enhancement. The reference system (Sys_0_) provides the baseline response, while the progressive incorporation of Si_3_N_4_, graphene, and ssDNA layers modifies the plasmonic behavior. The SPR reflectance curves in Figure 2a demonstrate a systematic shift in the resonance angle as additional layers are introduced. The blue curve (Sys_0_) exhibits an SPR peak at 68.6°, corresponding to the simplest prism-silver-plasma blood system. Replacing the sensing medium with normal (I) stage erythrocytes in Sys_1_ (orange curve) shifts the resonance to 78.2°, reflecting the higher refractive index of RBCs. The addition of Si_3_N_4_ in Sys_2_ (green curve) further displaces the peak to 84.2°. The resonance angle continues to increase with graphene integration in Sys_3_ (red curve) at 85.3°, while ssDNA functionalisation in Sys_4_ (purple curve) produces the highest shift at 86.2°.

The attenuation analysis in Figure 2b and Supplementary Table S1 indicates a progressive deepening of the SPR dip, correlating with enhanced plasmonic excitation. The reference system Sys_0_ exhibits minimal attenuation (0.02%), while Sys_1_ increases to 0.55%. A notable rise is observed in Sys_2_ (10.34%), confirming the effect of Si_3_N_4_ on resonance strength. The trend continues in Sys_3_ (35.45%) and Sys_4_ (45.37%), where graphene and ssDNA layers are expected to contribute to stronger energy transfer and enhanced molecular interaction. The FWHM results in Figure 2c provide insights into resonance sharpness, a critical factor for detection accuracy. Sys_0_ exhibits the narrowest value at 0.94°, while Sys_1_ broadens to 1.61°, indicating a slight reduction in resonance sharpness due to the increased refractive index of erythrocytes. The inclusion of Si_3_N_4_ in Sys_2_ results in a wider peak at 2.79°, while Sys_3_ and Sys_4_ further increase to 4.16° and 4.57°, respectively, demonstrating the effect of graphene and ssDNA layers on plasmonic curve broadening.

The sensitivity enhancement analysis in Figure 2d confirms the increasing detection capability of the biosensor. Sys_1_ achieves a 13.85% improvement over Sys_0_, followed by a significant increase in Sys_2_ (22.65%), reinforcing the role of Si_3_N_4_ in enhancing plasmonic response. Sys_3_ (24.30%) and Sys_4_ (25.60%) exhibit the highest sensitivity values, once more evidencing the positive influence of graphene and ssDNA functionalisation. Considering the superior performance observed in Sys_3_ and Sys_4_, these two configurations have been selected for further analysis. The enhanced resonance characteristics make them the most promising candidates for malaria biomarker detection, while the comparison between Sys_3_ and Sys_4_ allows for evaluating the specific contribution of the ssDNA layer.

With these results in mind, we point out the contribution of each material layer to the sensor’s performance, across five configurations (Sys_0_ to Sys_4_), as summarised in Table 1. Sys_0_ (non-malaria contribution) and Sys_1_, which rely solely on a silver layer, serve as reference systems. The sequential inclusion of silicon nitride (Sys_2_), graphene (Sys_3_), and ssDNA (Sys_4_) resulted in progressive modification in attenuation, FWHM, and sensitivity enhancement, as illustrated in Figure 2a–d. These results confirm that the enhanced plasmonic behavior and sensing performance observed in Sys_3_ and Sys_4_ are not due to the silver layer alone, but rather to the synergistic effects of the added Si_3_N_4_ and graphene layers, which improve field confinement and biomolecular interaction. The final ssDNA layer further supports sequence-specific biorecognition, enhancing diagnostic potential.

### 3.2 Metal thickness optimization

**FIGURE 3:**
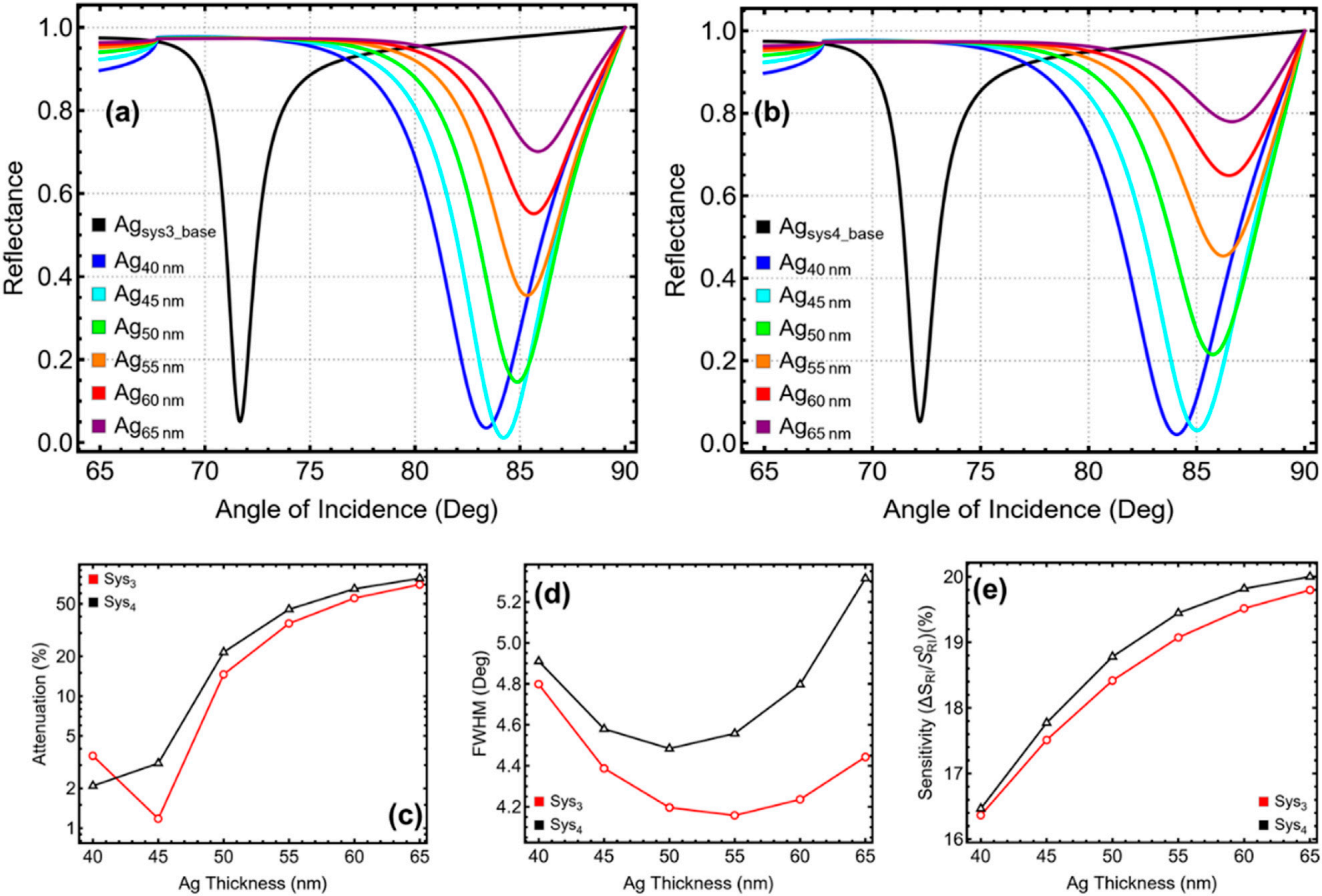
Performance analysis of Sys_3_ and Sys_4_ configurations with varying silver (Ag) thicknesses from 40 to 65 nm. **(a,b)** SPR reflectance curves for Sys_3_ and Sys_4_, respectively. **(c)** Percentage of attenuation for each configuration, y-axis-log scale is considered. **(d)** FWHM for each configuration. **(e)** Sensitivity enhancement (%) for Sys_3_ and Sys_4_, relative to the baseline systems constructed with initial parameters (Ag_sys3_base_ and Ag_sys4_base_).

Figure 3 and Supplementary Table S2 present the performance analysis of Sys_3_ and Sys_4_ by varying the silver (Ag) thickness from 40 to 65 nm. The results are analysed in relation to the baseline configurations (Ag_sys3_base_ and Ag_sys4_base_), represented in black, which correspond to the initial parameters reported in Table 2. The SPR curves in Figures 3a, b demonstrate the resonance shift across different silver thicknesses, while Figures 3c–e provide a quantitative comparison of attenuation, FWHM, and sensitivity enhancement. The reflectance curves indicate a systematic increase in the resonance angle as the silver thickness increases. The SPR peak for Sys_3_ shifts from 83.39° at 40 nm to 85.34° at 55 nm, beyond which the shift becomes less pronounced, reaching 85.85° at 65 nm (Figure 3a). A similar trend is observed in Sys_4_, where the resonance angle increases from 84.08° at 40 nm to 86.23° at 55 nm, stabilising at 86.63° at 65 nm. The largest shifts occur between 40 nm and 55 nm, confirming that plasmonic coupling improves significantly up to this range, while additional thickness beyond 55 nm contributes marginally to further shifting.

The attenuation analysis in Figure 3c reveals a notable increase in plasmonic absorption as silver thickness increases. At 40 nm, Sys_3_ and Sys_4_ exhibit low attenuation values of 3.53% and 2.09%, respectively, suggesting weaker plasmonic excitation. A substantial rise is observed at 50 nm, where attenuation reaches 14.60% in Sys_3_ and 21.51% in Sys_4_. The highest values occur at 65 nm, where Sys_3_ reaches 70.11% and Sys_4_ reaches 77.97%, indicating that excessive silver thickness results in stronger plasmonic absorption but also higher energy dissipation, which may negatively impact sensor performance. The FWHM results in Figure 3d indicate that moderate silver thicknesses improve resonance sharpness, with the lowest values recorded between 50 nm and 55 nm. In Sys_3_, FWHM decreases from 4.80° at 40 nm to 4.16° at 55 nm, before broadening again to 4.44° at 65 nm. A similar behaviour is observed in Sys_4_, where FWHM reduces from 4.91° at 40 nm to 4.56° at 55 nm, before widening to 5.32° at 65 nm. This pattern suggests that while thicker silver layers enhance resonance strength, excessive values result in broader spectral features, reducing measurement precision.

Sensitivity enhancement follows an increasing trend across all thicknesses, with the highest values observed at 65 nm (Figure 3d). In Sys_3_, sensitivity increases from 16.36% at 40 nm to 19.80% at 65 nm, while in Sys_4_, sensitivity improves from 16.47% at 40 nm to 20.0% at 65 nm. Despite this improvement, the broadening of the resonance peak at higher thicknesses compromises detection accuracy, indicating that sensitivity alone cannot determine the optimal configuration.

Considering a balance between a minimum attenuation, reasonable FWHM, and sensitivity enhancement, a silver thickness of 45 nm is selected for Sys_3_ and 40 nm is chosen for Sys_4_. In Sys_3_, 45 nm ensures a well-defined resonance with an FWHM of 4.39°, an attenuation of 1.18%, and a sensitivity enhancement of 17.51%, balancing plasmonic efficiency and detection accuracy. For Sys_4_, 40 nm is chosen due to its stable response with an FWHM of 4.91°, an attenuation of 2.09%, and a sensitivity enhancement of 16.47%, preventing excessive energy dissipation while maintaining effective resonance behaviour. These selections ensure that Sys_3_ and Sys_4_ maintain optimal plasmonic behaviour while preventing unnecessary signal degradation.

### 3.3 Silicon nitride thickness optimization

**FIGURE 4:**
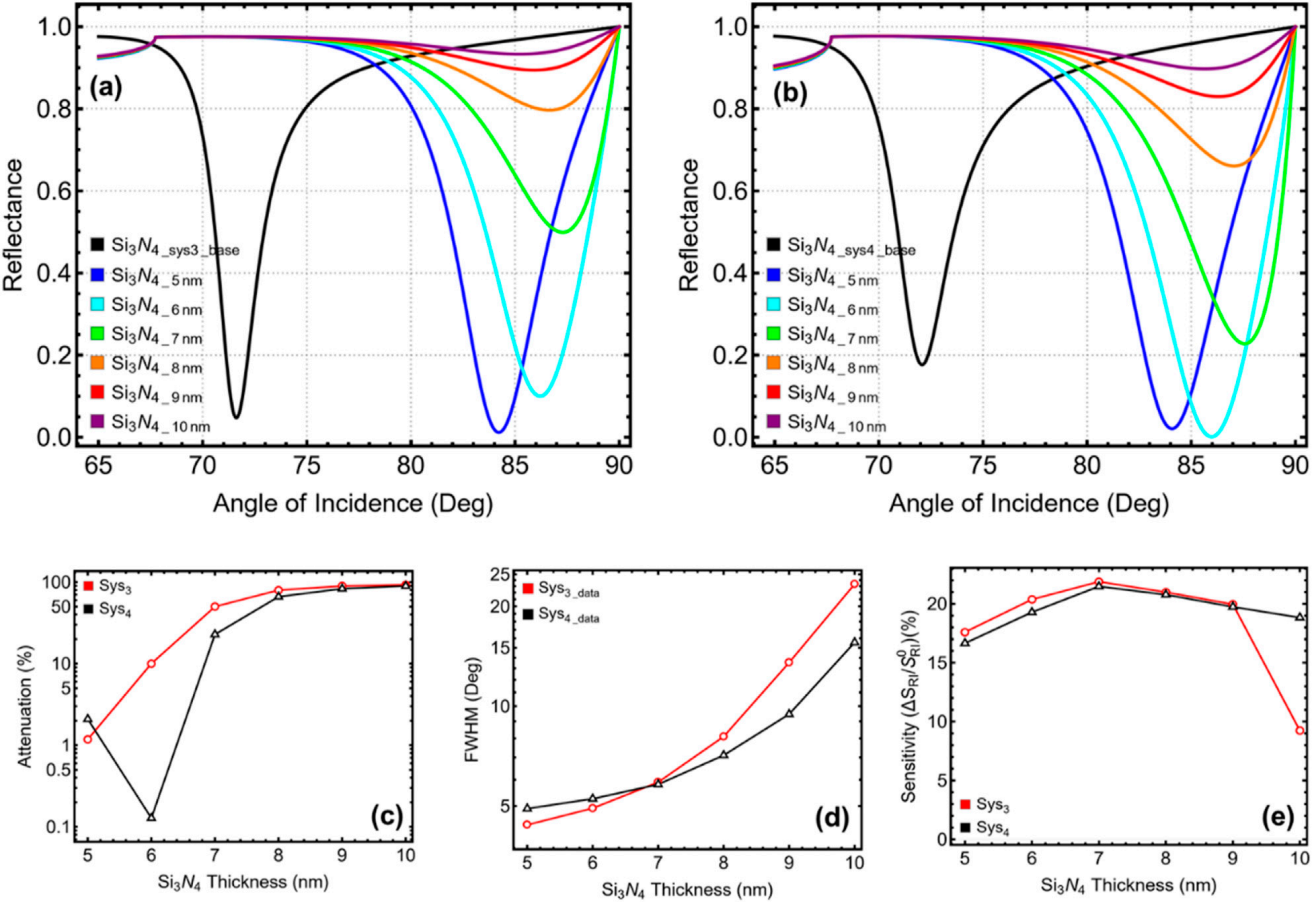
Performance analysis of Sys_3_ and Sys_4_ configurations with varying silicon nitride thicknesses from 5 to 10 nm. **(a,b)** SPR reflectance curves for Sys_3_ and Sys_4_, respectively. **(c)** Percentage of attenuation for each configuration, y-axis-log scale is considered. **(d)** FWHM for each configuration, y-axis-log scale is considered. **(e)** Sensitivity enhancement (%) for Sys_3_ and Sys_4_, relative to the baseline systems constructed with initial parameters (Si_3_N_4_sys3_base_ and Si_3_N_4_sys4_base_) and optimized silver thickness value.

The influence of silicon nitride (Si_3_N_4_) thickness on the plasmonic behaviour of Sys_3_ and Sys_4_ is analysed in Figure 4 and Supplementary Table S3, considering values from 5 to 10 nm while maintaining the previously optimized silver thickness of 45 nm for Sys_3_ and 40 nm for Sys_4_. The baseline configurations (Si_3_N_4_sys3_base_ and Si_3_N_4_sys4_base_), represented in black, serve as reference systems for comparison. The reflectance curves in Figure 4a (Sys_3_) and Figure 4b (Sys_4_) show a progressive shift in resonance angle with increasing Si_3_N_4_ thickness, confirming that additional dielectric material modifies plasmonic confinement. In Sys_3_, the SPR peak moves from 84.22° at 5 nm to 87.29° at 7 nm, before slightly decreasing at larger thicknesses. Similarly, Sys_4_ exhibits a resonance shift from 84.08° at 5 nm to a maximum of 87.56° at 7 nm, beyond which the angle stabilises. These results suggest that moderate Si_3_N_4_ thickness effectively enhances plasmonic interaction, but further increase beyond 7 nm contributes marginally to resonance tuning. The attenuation trends, illustrated in Figure 4c, indicate a rapid increase in plasmonic absorption with increasing Si_3_N_4_ thickness. At 5 nm, Sys_3_ exhibits a low attenuation of 1.18%, which rises to 49.92% at 7 nm. A similar behaviour is observed in Sys_4_, where attenuation increases from 2.09% at 5 nm to 22.78% at 7 nm. Beyond this thickness, the attenuation continues rising sharply, reaching 92.82% in Sys_3_ and 89.72% in Sys_4_ at 10 nm, confirming that excessive Si_3_N_4_ thickness leads to excessive energy dissipation, diminishing the efficiency of the SPR response.

The FWHM results in Figure 4d reveal that thinner Si_3_N_4_ layers provide sharper resonance peaks, with the lowest values recorded at 5 nm for Sys_3_ (4.39°) and 6 nm for Sys_4_ (5.26°). Beyond these thicknesses, FWHM progressively increases, reaching 5.90° at 7 nm in Sys_3_ and 5.81° in Sys_4_, and continuing to broaden at larger values, reaching 15.56° at 10 nm for Sys_4_ and 23.33° for Sys_3_. This degradation in resonance sharpness suggests that increasing Si_3_N_4_ thickness beyond optimal values results in excessive plasmon damping, reducing detection precision. The sensitivity enhancement, as shown in Figure 4e, follows a nonlinear trend, peaking at 7 nm before slightly decreasing at larger thicknesses. In Sys_3_, sensitivity rises from 17.60% at 5 nm to a maximum of 21.89% at 7 nm, before dropping at 10 nm (9.24%). Similarly, in Sys_4_, sensitivity increases from 16.65% at 5 nm to 21.49% at 7 nm, before decreasing at 10 nm (18.83%). These results confirm that while Si_3_N_4_ enhances plasmonic interaction, excessive thickness degrades sensitivity and accuracy.

Considering the balance between SPR peak shift, attenuation, resonance sharpness, and sensitivity, a Si_3_N_4_ thickness of 5 nm is selected for Sys_3_ and 6 nm for Sys_4_. The 5 nm thickness in Sys_3_ provides a well-defined resonance with a narrow FWHM of 4.39°, minimal attenuation of 1.18%, and a sensitivity enhancement of 17.60%. In Sys_4_, 6 nm achieves an optimal trade-off, yielding an FWHM of 5.26°, low attenuation of 0.13%, and a sensitivity improvement of 19.29%, maintaining strong resonance quality while preventing excessive energy dissipation.

### 3.4 Number of 2D nanolayers optimization

**FIGURE 5:**
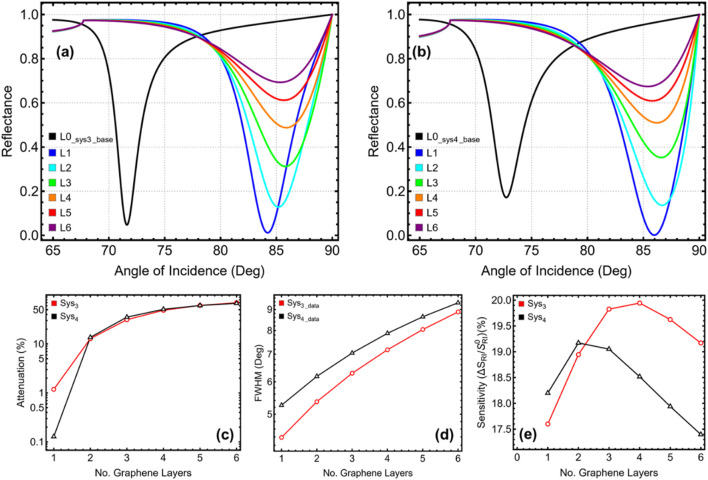
Performance analysis of Sys_3_ and Sys_4_ configurations with varying the number of graphene layers from 1 (L1) to 6 (L6). **(a,b)** SPR reflectance curves for Sys_3_ and Sys_4_, respectively. **(c)** Percentage of attenuation for each configuration, y-axis-log scale is considered. **(d)** FWHM for each configuration. **(e)** Sensitivity enhancement (%) for Sys_3_ and Sys_4_, relative to the baseline systems constructed with initial parameters (L0__sys3_base_ and L0__sys4_base_) and optimized silver/silicon nitride thickness values.

The effect of varying the number of graphene layers from 1 (L1) to 6 (L6) on the sensor behaviour of Sys_3_ and Sys_4_ is analysed in Figure 5 and Supplementary Table S4, following the previously optimized silver and Si_3_N_4_ thicknesses of 45 nm and 5 nm for Sys_3_, and 40 nm and 6 nm for Sys_4_, respectively. The baseline configurations (L0__sys3_base_ and L0__sys4_base_), represented in black, serve as reference systems for comparison. The reflectance curves in Figure 5a (Sys_3_) and Figure 5b (Sys_4_) illustrate a progressive shift in resonance angle with increasing graphene layers, confirming its role in modulating plasmonic sensor behaviour. In Sys_3_, the SPR peak shifts from 84.22° at L1 to 85.81° at L3, beyond which the shift becomes negligible, stabilising at 85.341° at L6. Similarly, Sys_4_ exhibits a resonance shift from 85.98° at L1 to 86.68° at L2, before slightly reducing at 85.39° at L6. The most significant shifts occur between L1 and L3 in Sys_3_, and L1 and L2 in Sys_4_, suggesting that additional graphene layers enhance plasmonic interaction up to a certain threshold, beyond which further increase provides diminishing returns.

The attenuation results in Figure 5c reveal a continuous increase in plasmonic absorption with additional graphene layers. At L1, Sys_3_ exhibits an attenuation of 1.18%, which rises to 12.82% at L2 and 31.22% at L3. A similar trend is observed in Sys_4_, where attenuation increases from 0.13% at L1 to 13.62% at L2, then reaching 35.25% at L3. Beyond these values, attenuation continues increasing, reaching 69.31% at L6 in Sys_3_ and 67.42% in Sys_4_, confirming that excessive graphene layers lead to higher losses, reducing energy efficiency. The FWHM analysis in Figure 5d highlights a progressive broadening of the resonance curve with more graphene layers. In Sys_3_, FWHM increases from 4.39° at L1 to 6.29° at L3, reaching 8.89° at L6, while in Sys_4_, it rises from 5.26° at L1 to 6.18° at L2, then continues broadening to 9.345° at L6. This trend indicates that while graphene enhances resonance strength, excessive layers lead to spectral broadening, negatively impacting detection accuracy. The sensitivity enhancement, illustrated in Figure 5e, exhibits a nonlinear trend, peaking at L3 in Sys_3_ and L2 in Sys_4_ before decreasing at larger values. In Sys_3_, sensitivity improves from 17.60% at L1 to 19.83% at L3, before reducing to 19.17% at L6. A similar behaviour is observed in Sys_4_, where sensitivity increases from 18.20% at L1 to 19.17% at L2, before declining to 17.40% at L6. These results confirm that moderate graphene layers enhance molecular interactions, while excessive layers reduce sensitivity due to increased optical losses.

To achieve an optimal balance between attenuation, spectral sharpness, and sensitivity, 2 graphene layers are selected for both Sys_3_ and Sys_4_. Particularly, after 2 layers the attenuation (>30%) and FWHM (>6°) values critically increase. In Sys_3_, L2 ensures a well-defined resonance with an FWHM of 5.36°, moderate attenuation of 12.82%, and a sensitivity enhancement of 18.95%, ensuring strong plasmonic coupling without excessive spectral broadening. In Sys_4_, L2 achieves an optimal trade-off, providing an FWHM of 6.18°, controlled attenuation of 13.62%, and a sensitivity improvement of 19.17%, ensuring efficient detection performance. Although increasing graphene layers beyond L3 enhances sensitivity, it also leads to broader resonance peaks and higher losses, reducing detection precision.

### 3.5 ssDNA thickness optimization

**FIGURE 6:**
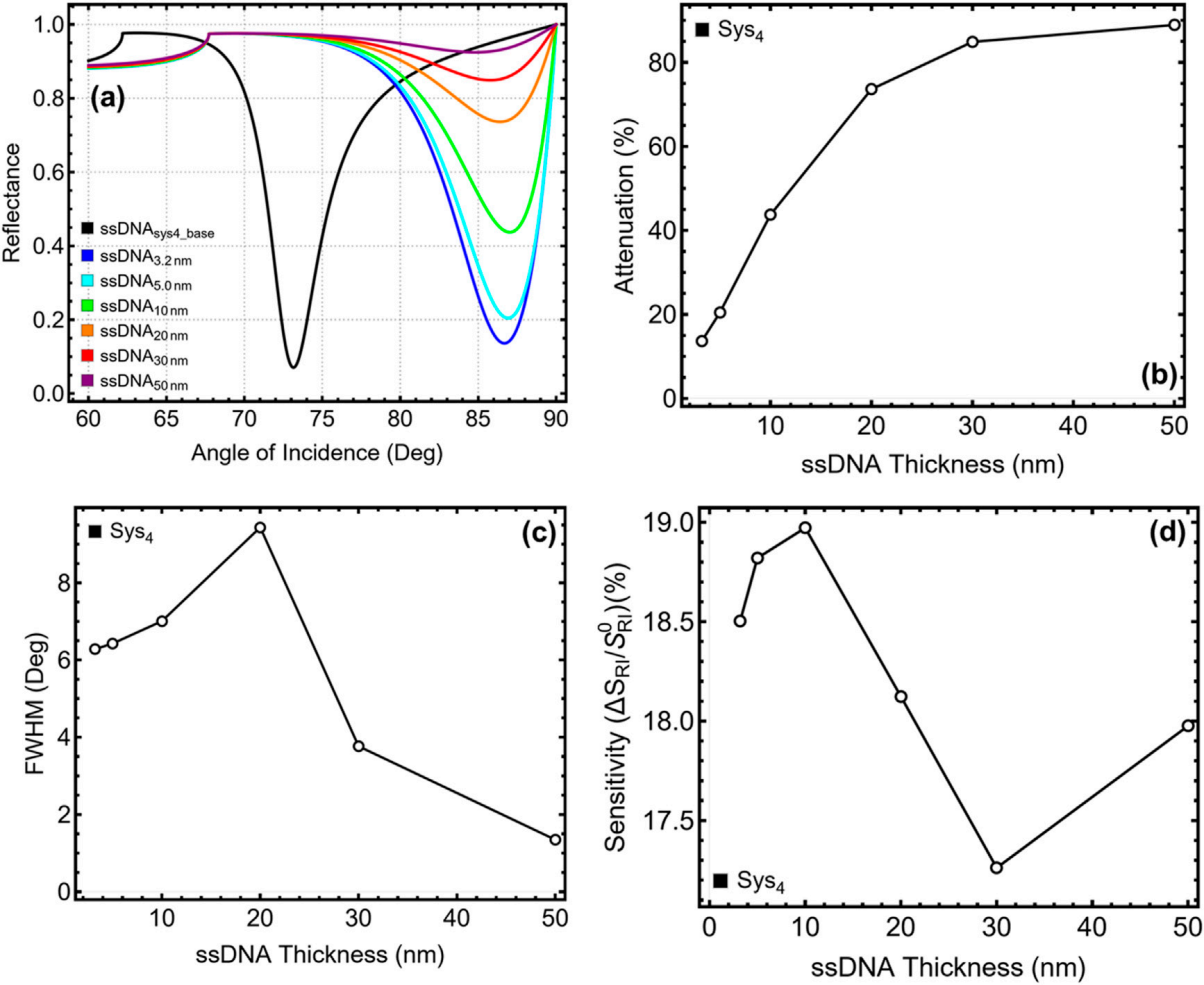
Performance analysis of Sys_4_ configuration with varying the ssDNA layer thickness from 3.2 nm to 50 nm. **(a)** SPR reflectance curves. **(b)** Percentage of attenuation for each configuration. **(c)** FWHM for each configuration. **(d)** Sensitivity enhancement (%), relative to the baseline system constructed with initial parameters (ssDNA_sys4_base_) and optimized silver/silicon nitride/graphene values.

The influence of ssDNA thickness on the plasmonic sensor response of Sys_4_ is analysed in Figure 6 and Supplementary Table S5, considering values from 3.2 nm to 50 nm, while maintaining the previously optimized silver, Si_3_N_4_, and graphene layers at 40 nm, 6 nm, and 2 layers, respectively. Since Sys_4_ is the only system incorporating the ssDNA layer, the analysis is exclusive to this configuration. The baseline system (ssDNA__sys4_base_), represented in black, is used as a reference. The reflectance curves in Figure 6a illustrate a systematic shift in resonance angle as ssDNA thickness increases, confirming its role in modulating plasmonic interaction. The SPR peak moves from 86.68° at 3.2 nm to 87.03° at 10 nm, beyond which a progressive decrease occurs, reaching 85.52° at 50 nm. The most pronounced shifts are observed between 3.2 nm and 10 nm, suggesting that thinner ssDNA layers significantly enhance molecular binding efficiency, while further increase leads to a less effective refractive index contrast, reducing the resonance shift.

The attenuation results in Figure 6b indicate a strong correlation between ssDNA thickness and plasmonic absorption. At 3.2 nm, Sys_4_ exhibits an attenuation of 13.62%, which increases to 20.45% at 5 nm and 43.71% at 10 nm. Beyond 10 nm, attenuation continues rising sharply, reaching 88.93% at 50 nm, confirming that excessive ssDNA thickness introduces considerable energy dissipation, reducing overall sensor stability. The FWHM analysis in Figure 6c reveals that moderate ssDNA thickness contributes to sharp resonance peaks, while excessive thickness causes substantial spectral broadening. The lowest values are observed at 30 nm (3.77°) and 50 nm (1.35°), but these configurations exhibit severe attenuation, compromising their practical applicability. At 5 nm, FWHM remains controlled at 6.43°, ensuring a well-defined resonance curve, while higher thickness values increase broadening, reaching 9.44° at 20 nm before narrowing due to excessive losses. These results suggest that moderate ssDNA thickness provides the best trade-off between plasmonic confinement and spectral resolution.

Sensitivity enhancement, presented in Figure 6d, follows a nonlinear trend, peaking at 10 nm before decreasing at larger thicknesses. Sensitivity increases from 18.50% at 3.2 nm to 18.97% at 10 nm, but beyond this point, a gradual decline is observed, with values dropping to 17.98% at 50 nm. This trend confirms that ssDNA effectively enhances molecular binding up to a specific thickness, beyond which additional layers do not contribute significantly to detection sensitivity. To achieve an optimal balance between SPR peak position, attenuation, spectral sharpness, and sensitivity, 5 nm is selected as the optimal ssDNA thickness for Sys_4_. This choice is justified by its well-defined resonance peak (86.92°), moderate attenuation (20.45%), and a controlled FWHM of 6.43°, ensuring efficient plasmonic interaction without excessive losses.

### 3.6 Malaria detection

Supplementary Table S6 presents the refractive index and optimized thickness values for the final configurations of Sys_3_ and Sys_4_, alongside the refractive index values for different malaria stages. These parameters define the structural and optical characteristics of the optimized systems, which will now be tested against ring (II), trophozoite (III), and schizont (IV) stages of the malaria parasite to evaluate their detection performance.

Notably, the optimized configuration of Sys_3_ consists of a silver layer with a thickness of 45.0 nm, a silicon nitride layer of 5.0 nm, and a graphene layer corresponding to 0.34 nm multiplied by two layers. The refractive indices for these materials are 1.5151 for BK7 glass, 2.0394 for silicon nitride, and 3.0 + 1.1491i for graphene, while silver exhibits a refractive index of 0.056253 + 4.2760i. The structure of Sys_4_ is similar to Sys_3_ but incorporates an additional ssDNA layer for enhanced biomolecular interaction. The silver thickness is 40.0 nm, silicon nitride is 6.0 nm, and graphene remains at 0.34 nm per layer for two layers, while the ssDNA layer has a thickness of 5.0 nm with a refractive index of 1.462. Additionally, Supplementary Table S6 provides the refractive indices of the three malaria stages under investigation. The ring stage exhibits the highest refractive index at 1.395, followed by the trophozoite stage at 1.381, and the schizont stage at 1.371, confirming a progressive reduction in refractive index as the parasite matures. This variation in refractive index obviously is expected to influence the plasmonic response of the biosensor, allowing for differentiation between malaria stages based on resonance shifts.

**FIGURE 7:**
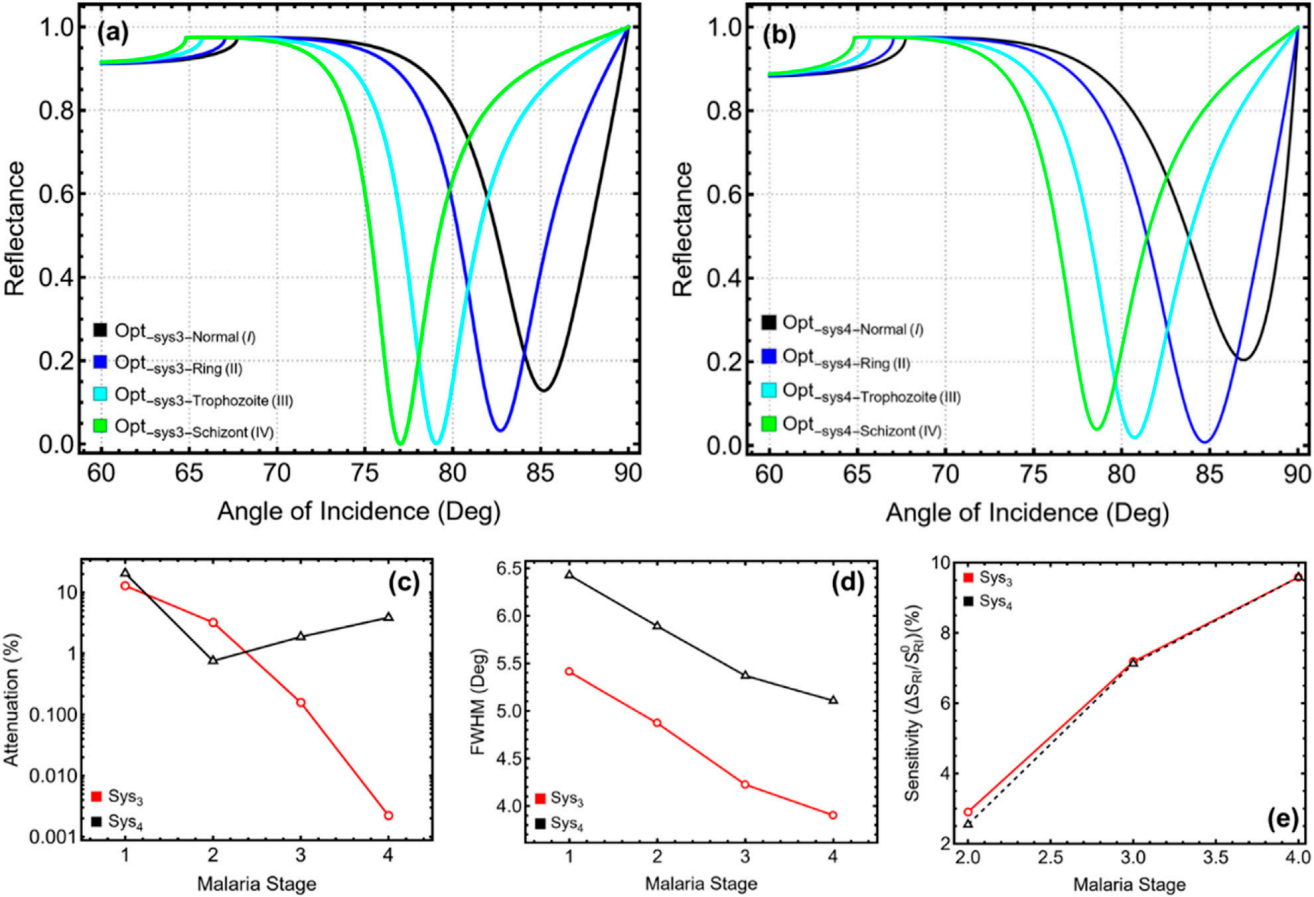
Performance analysis of optimized Sys_3_ and Sys_4_ configurations with varying the Malaria stage from Normal (I) to Schizont (IV). **(a,b)** SPR reflectance curves for optimized Sys_3_ and Sys_4_, respectively. **(c)** Percentage of attenuation for each configuration, y-axis-log scale is considered. **(d)** FWHM for each configuration. **(e)** Sensitivity enhancement (%) for Sys_3_ and Sys_4_, relative to the optimized systems taking as a sensing medium the Normal (I) stage (Opt__sys3-Normal(I)_ and Opt__sys4-Normal(I)_).

Figure 7 and Supplementary Table S7 present the performance analysis of the optimized Sys_3_ and Sys_4_ configurations when exposed to different malaria stages, ranging from normal (I) erythrocytes to schizont (IV) stage-infected cells. The baseline configurations (Opt__sys3_Normal(I)_ and Opt__sys4_Normal(I)_), represented in black, serve as reference systems. The reflectance curves in Figure 7a (Sys_3_) and Figure 7b (Sys_4_) demonstrate a progressive shift in resonance angle as the malaria stage transitions from normal to schizont-infected erythrocytes, consistent with the decreasing refractive index of infected cells. In Sys_3_, the SPR peak moves from 85.18° for normal erythrocytes to 82.71° for the ring stage, 79.07° for the trophozoite stage, and 77.02° for the schizont stage, indicating a significant resonance shift across infection stages. Similarly, Sys_4_ exhibits a shift from 86.92° for normal erythrocytes to 84.71° for the ring stage, 80.73° for the trophozoite stage, and 78.59° for the schizont stage, reinforcing the system’s ability to detect malaria progression.

The attenuation results in Figure 7c reveal a decreasing trend as the malaria stage progresses, correlating with the reduced optical confinement associated with lower refractive index values. For Sys_3_, attenuation drops from 12.82% at the normal stage to 3.21% at the ring stage, 0.16% at the trophozoite stage, and a negligible 0.002% at the schizont stage. In Sys_4_, attenuation follows a similar pattern, decreasing from 20.45% for normal erythrocytes to 0.75% for the ring stage, 1.86% for the trophozoite stage, and 3.86% for the schizont stage. The spectral width analysis in Figure 7d indicates progressively narrower resonance peaks as malaria progresses, suggesting an improvement in detection resolution for later infection stages. In Sys_3_, the spectral width decreases from 5.41° for normal erythrocytes to 4.87° at the ring stage, 4.23° at the trophozoite stage, and 3.90° at the schizont stage, implying that infected cells enhance spectral precision due to their distinct refractive index properties. A similar trend is observed in Sys_4_, where the spectral width reduces from 6.43° at the normal stage to 5.89° at the ring stage, 5.37° at the trophozoite stage, and 5.11° at the schizont stage, ensuring highly defined resonance responses.

The sensitivity enhancement results in Figure 7e confirm a steady increase across malaria stages, highlighting the system’s ability to differentiate infected cells. In Sys_3_, sensitivity increases from 0.0% at the normal stage to 2.90% at the ring stage, 7.17% at the trophozoite stage, and 9.58% at the schizont stage, indicating progressively higher sensitivity to later-stage infections. Similarly, in Sys_4_, sensitivity rises from 0.0% for normal erythrocytes to 2.54% for the ring stage, 7.12% for the trophozoite stage, and 9.58% for the schizont stage. These results validate that both Sys_3_ and Sys_4_ effectively track malaria progression, demonstrating clear resonance shifts and increasing sensitivity enhancement with advancing infection stages.

To emphasize, the observed shift of the SPR resonance angle toward lower values with malaria (Figure 7a, b) progression is attributed to the decreasing refractive index of infected red blood cells. As the infection advances from ring to schizont stage, hemoglobin consumption and structural degradation reduce the optical density of the sensing medium. This decrease in refractive index lowers the momentum required for plasmon excitation, resulting in a leftward shift of the resonance angle.

### 3.7 Performance sensing metrics

**FIGURE 8:**
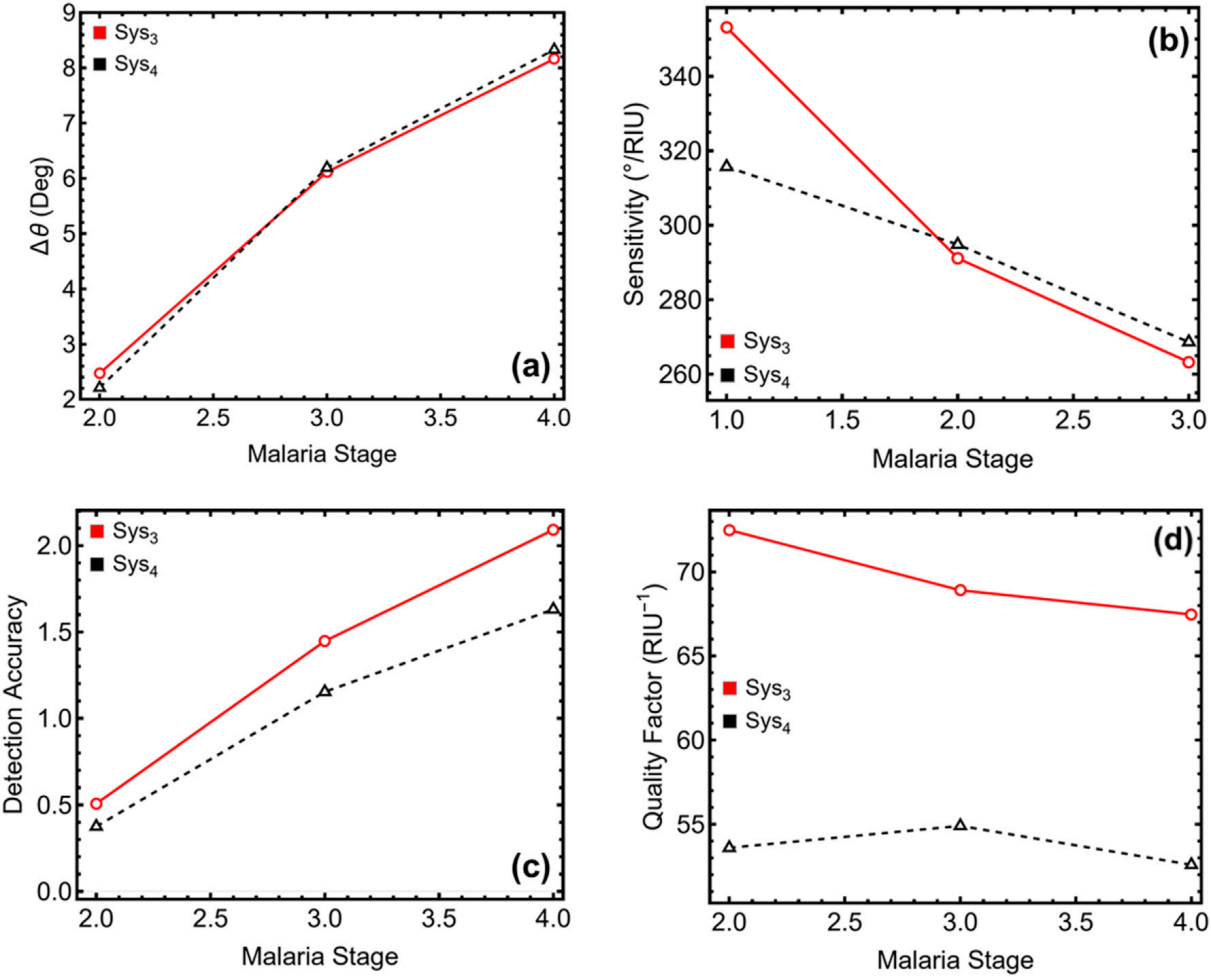
Performance metrics of the SPR biosensor for different malaria stages. **(a)** Variation in resonance angle shift (Δθ), **(b)** Sensitivity (°/RIU) to refractive index changes, **(c)** Detection accuracy, and **(d)** Quality factor (RIU^−1^) for each malaria stage.

Figure 8 and Table 3 present the key performance metrics for optimized Sys_3_ and Sys_4_ across different malaria stages, assessing their effectiveness in detecting infected erythrocytes based on variations in resonance angle shift, sensitivity to refractive index changes, detection accuracy, and quality factor. The resonance angle shift analysis in Figure 8a demonstrates a progressive increase in shift magnitude as malaria progresses. In Sys_3_, the resonance angle shift increases from 2.47° at the ring stage to 6.11° at the trophozoite stage and 8.16° at the schizont stage, while Sys_4_ exhibits shifts of 2.21°, 6.19°, and 8.33° for the same stages, respectively. The larger shifts observed in later stages indicate higher biomolecular adsorption, reinforcing the effectiveness of both systems in malaria detection. Sensitivity trends in Figure 8b indicate a decreasing response with advancing infection stages, corresponding to the declining refractive index of infected erythrocytes. For Sys_3_, sensitivity decreases from 353.14°/RIU at the ring stage to 291.14°/RIU at the trophozoite stage and 263.26°/RIU at the schizont stage, while Sys_4_ follows a similar trend, reducing from 315.71°/RIU to 294.81°/RIU and 268.65°/RIU. Although Sys_3_ exhibits slightly higher sensitivity, both configurations maintain strong plasmonic responses, ensuring reliable malaria detection.

**TABLE 3:** Numerical values of the SPR biosensor performance metrics for different malaria stages, corresponding to the results presented in Figure 8. Δθ represents the resonance angle shift, S is the sensitivity, DA is the detection accuracy, and QF is the quality factor.

Malaria stage	∆θ	*S* ( °/RIU )	DA	QF (*RIU* ^−1^)
Opt-Sys_3_				
Ring (II)	2.472	353.143	0.507	72.493
Trophozoite (III)	6.114	291.143	1.447	68.906
Schizont (IV)	8.161	263.258	2.091	67.465
Opt-Sys_4_				
Ring (II)	2.210	315.714	0.375	53.603
Trophozoite (III)	6.191	294.810	1.153	54.907
Schizont (IV)	8.328	268.645	1.630	52.594

Detection accuracy results in Figure 8c show a steady increase across malaria stages, highlighting improved detection capabilities for later infection stages. In Sys_3_, accuracy increases from 0.51 at the ring stage to 1.45 at the trophozoite stage and 2.09 at the schizont stage, while Sys_4_ follows a similar pattern, rising from 0.38 to 1.15 and 1.63. These results confirm that both configurations effectively distinguish infected cells, with Sys_3_ exhibiting higher accuracy due to its sharper resonance features. Quality factor analysis in Figure 8d reveals a declining trend with malaria progression, consistent with the increasing spectral broadening and attenuation observed in previous analyses. In Sys_3_, the quality factor decreases from 72.49 RIU^−1^ at the ring stage to 68.91 RIU^−1^ at the trophozoite stage and 67.47 RIU^−1^ at the schizont stage, while Sys_4_ follows a similar trend, dropping from 53.60 RIU^−1^ to 54.91 RIU^−1^ and 52.59 RIU^−1^. Despite the reduction, the values remain within an acceptable range for biosensing applications, ensuring high-quality resonance responses. While Sys_3_ exhibits higher sensitivity and quality factor, Sys_4_ benefits from improved biorecognition due to the ssDNA layer, making both configurations highly suitable for malaria biomarker detection applications.

The higher QF observed in Sys_3_ compared to Sys_4_ is primarily due to its narrower resonance width. While both systems exhibit similar sensitivity values, the inclusion of the ssDNA layer in Sys_4_ introduces additional optical damping and broadening of the resonance dip. This effect slightly reduces angular precision and results in lower QF values for Sys_4_, despite its enhanced biorecognition capabilities.

**FIGURE 9:**
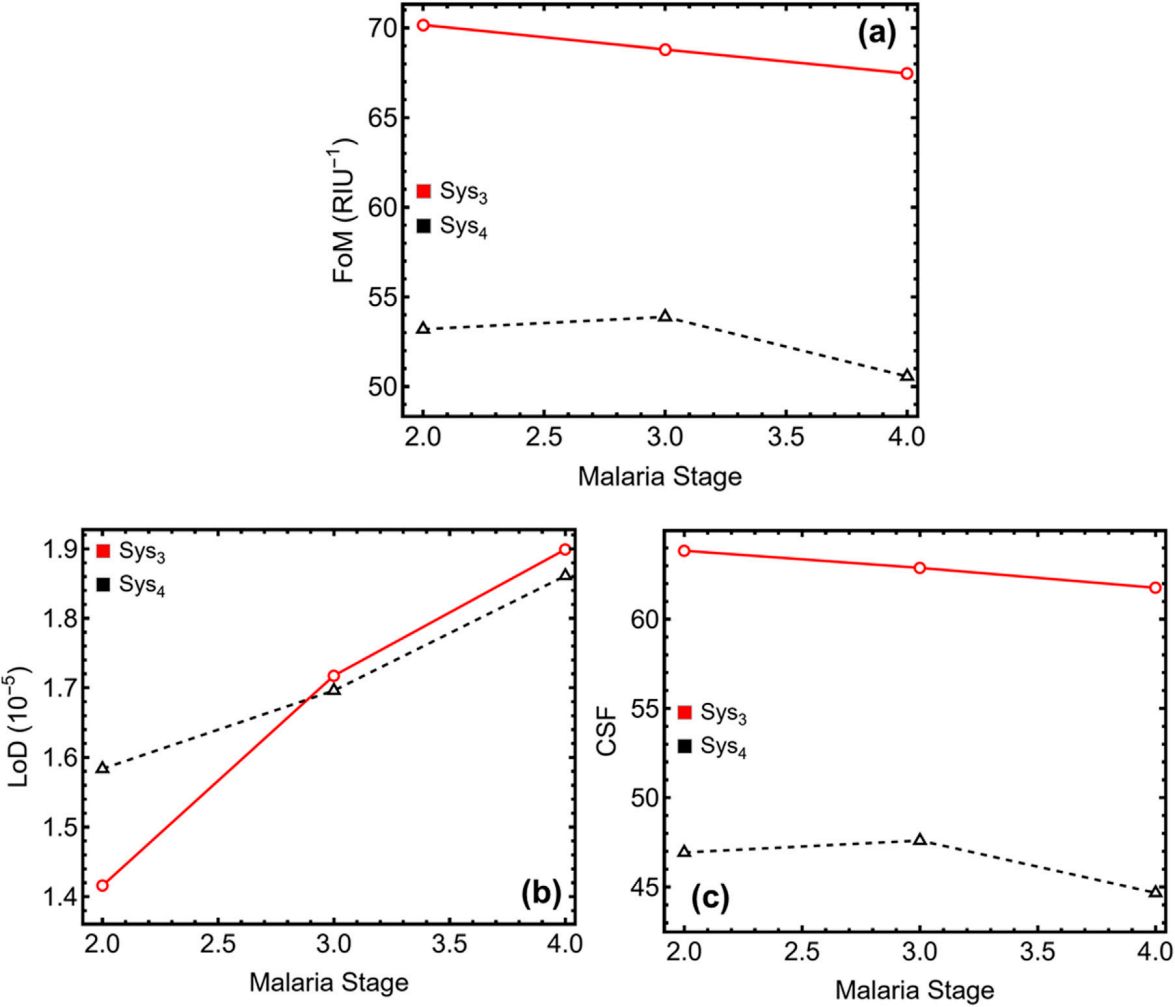
Additional performance metrics of the SPR biosensor for different malaria stages. **(a)** Figure of merit (FoM), **(b)** Limit of detection (LoD), and **(c)** Comprehensive sensitivity factor (CSF).

Figure 9 and Table 4 present the further evaluation of key sensor quality parameters for Sys_3_ and Sys_4_, including the figure of merit, limit of detection, and comprehensively sensitive factor, across different malaria stages. The results in Figure 9a illustrate a declining figure of merit as malaria progresses, indicating a slight reduction in sensing efficiency due to spectral broadening and variations in plasmonic confinement. In Sys_3_, the figure of merit decreases from 70.16 RIU^−1^ at the ring stage to 68.80 RIU^−1^ at the trophozoite stage and 67.46 RIU^−1^ at the schizont stage, while Sys_4_ exhibits lower values, reducing from 53.20 RIU^−1^ at the ring stage to 53.88 RIU^−1^ at the trophozoite stage. Despite the decline, Sys_3_ consistently outperforms Sys_4_, maintaining higher plasmonic efficiency across all malaria stages. The limit of detection results in Figure 9b reveal an increasing trend with malaria progression, signifying improved detection capability in later infection stages. In Sys_3_, the limit of detection rises from 1.42 × 10^−5^ at the ring stage to 1.72 × 10^−5^ at the trophozoite stage and 1.90 × 10^−5^ at the schizont stage, while Sys_4_ follows a similar pattern, increasing from 1.58 × 10^−5^ at the ring stage to 1.70 × 10^−5^ at the trophozoite stage. These findings confirm that both configurations achieve excellent detection sensitivity, with Sys_3_ demonstrating a slightly enhanced ability to detect refractive index variations due to its sharper resonance features.

**TABLE 4:** Numerical values of the additional SPR biosensor performance metrics for different malaria stages, corresponding to the results presented in Figure 9. FoM represents the figure of merit, LoD is the limit of detection, and CSF is the comprehensive sensitivity factor.

Malaria stage	FoM (*RIU* ^−1^)	LoD (10^–5^)	CSF
Opt-Sys_3_			
Ring (II)	70.163	1.415	63.846
Trophozoite (III)	68.797	1.717	62.883
Schizont (IV)	67.464	1.899	61.764
Opt-Sys_4_			
Ring (II)	53.199	1.583	46.936
Trophozoite (III)	53.883	1.696	47.602
Schizont (IV)	50.565	1.861	44.675

The comprehensively sensitive factor, depicted in Figure 9c, assesses the biosensor’s combined sensitivity, selectivity, and stability, providing an overall performance metric. In Sys_3_, this factor decreases from 63.85 at the ring stage to 62.88 at the trophozoite stage and 61.76 at the schizont stage, while Sys_4_ exhibits lower values, reducing from 46.94 to 47.60 across the same stages. Again, these results indicate that Sys_3_ maintains slightly superior overall efficiency, benefiting from a more defined resonance response and a higher figure of merit. Although Sys_4_ offers enhanced biorecognition due to the ssDNA functionalisation, Sys_3_ remains the optimal configuration for precise malaria stage differentiation, ensuring higher detection efficiency, lower detection limits, and improved sensing stability.

### 3.8 State-of-the-art comparison

Table 5 presents a comparative analysis of the sensitivity performance of Sys_3_ and Sys_4_ against previously reported surface plasmon resonance biosensors, demonstrating the superior sensing capabilities of the proposed configurations. Unlike previous works, which primarily report a single sensitivity value without stage differentiation, the current study provides a detailed evaluation across different malaria infection stages, offering a more comprehensive insight into biosensor performance.

**TABLE 5:** Comparison with state-of-the-art SPR biosensors.

Configuration	*S* ( °/RIU )	Ref. #
TiO_2_/Ag/MoSe/Graphene	194.0	Moznuzzaman et al. (2021)
Au/PtSe_2_/Graphene	200.0	Mostufa et al. (2021)
TiO_2_/ZnO/Au/MoS_2_/GO	210.75	Guo et al. (2020)
Rh/Ag/Si/Graphene	220.0	Mishra and Mishra (2016)
Au/MXene/Au/Graphene (Trophozoite (III))	258.28	Karki et al. (2024)
Ag/Si_3_N_4_/Graphene (Sys_3_)	353.14 (II) 291.14 (III) 263.26 (IV)	This work
Ag/Si_3_N_4_/Graphene/ssDNA (Sys_4_)	315.71 (II) 294.81 (III) 268.65 (IV)	This work

The reported values from the literature indicate sensitivity ranging from 194.0°/RIU for TiO_2_/Ag/MoSe_2_/Graphene to a maximum of 258.28°/RIU for Au/MXene/Au/Graphene targeting the trophozoite stage. Other configurations, such as TiO_2_/ZnO/Au/MoS_2_/GO, Rh/Ag/Si/Graphene, and Au/PtSe_2_/Graphene, exhibit sensitivity values between 200.0°/RIU and 220.0°/RIU, highlighting the typical range observed in conventional multi-layered plasmonic biosensors. In contrast, the optimized configurations in this work significantly outperform previously reported values, achieving 353.14°/RIU for the ring stage, 291.14°/RIU for the trophozoite stage, and 263.26°/RIU for the schizont stage in Sys_3_. Similarly, Sys_4_ achieves 315.71°/RIU for the ring stage, 294.81°/RIU for the trophozoite stage, and 268.65°/RIU for the schizont stage, confirming enhanced sensitivity compared to all previously investigated systems.

A key distinction of this work is the explicit differentiation of malaria infection stages, which has not been reported in most prior studies. The only reference providing stage-specific sensitivity (Au/MXene/Au/Graphene at 258.28°/RIU for the trophozoite stage) falls significantly below the corresponding values achieved by Sys_3_ (291.14°/RIU) and Sys_4_ (294.81°/RIU), reinforcing the superior performance of the proposed configurations. These findings confirm that the integration of silver, silicon nitride, graphene, and ssDNA layers in Sys_3_ and Sys_4_ results in a substantial enhancement in plasmonic sensitivity, enabling a more accurate and stage-specific detection of malaria infections.

We point out that the ability to differentiate malaria stages based on resonance angle shifts provides a clinically valuable feature beyond mere detection. Early-stage identification (e.g., ring stage) supports timely intervention, which is critical for reducing disease severity and transmission. In contrast, monitoring progression to later stages such as trophozoite and schizont enables assessment of treatment efficacy and disease resolution. Stage-specific detection may also assist in stratifying patient risk, adjusting therapeutic regimens, and evaluating drug resistance by correlating parasitic load with optical response, offering a practical tool for both diagnosis and longitudinal care.

### 3.9 Potential fabrication

To further emphasize, this study is entirely theoretical and based on the optical modeling of multilayer structures using refractive index values reported in the literature. As such, no specific fabrication method was employed. However, the proposed SPR configuration incorporates two layers of graphene, a structure that is feasible using techniques such as chemical vapor deposition (CVD) (Ning et al., 2025), which is widely used for producing large-area, uniform graphene films. Although practical challenges related to graphene transfer, contamination, and reproducibility are acknowledged, the present simulations assume an idealized, defect-free graphene interface to evaluate the biosensor’s theoretical performance potential.

**FIGURE 10:**
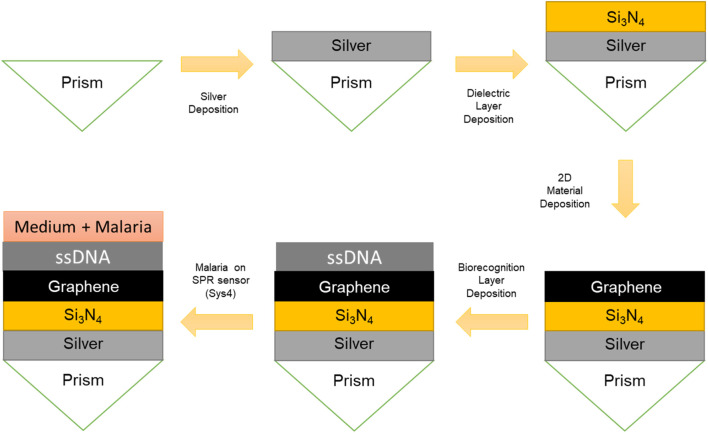
Schematic representation of the potential fabrication process of the proposed Sys_4_ SPR sensor.

With this in mind, a schematic of the potential fabrication process for the Sys_4_ SPR biosensor configuration is presented in Figure 10. The process begins with the deposition of a thin silver film onto the base of the prism. This layer can be deposited using physical vapor deposition techniques such as thermal evaporation (Mohammed et al., 2018) or magnetron sputtering (Hajakbari and Ensandoust, 2016), which offer good film uniformity and adhesion to glass substrates. A dielectric interlayer of silicon nitride is then deposited onto the silver surface. This step can be performed via plasma-enhanced chemical vapor deposition (PECVD) (Wardenberg et al., 2025) or atomic layer deposition (ALD) (Kim et al., 2024), both of which provide high control over thickness at the nanometer scale.

Subsequently, bilayer graphene is transferred onto the Si_3_N_4_ surface. This is typically achieved by first synthesizing monolayer graphene on copper substrates via CVD (Liu et al., 2024), followed by a wet transfer process using a polymer support film such as polymethyl methacrylate (PMMA) (Deokar et al., 2015). After transfer, the support is removed, and the graphene is cleaned and left on the dielectric interface. While the modeling in this study assumes an ideal graphene surface, this step may introduce interfacial residues or bubbles that could affect practical sensor performance and would require optimization in future experimental work.

The final step involves the immobilization of a thiol-tethered ssDNA layer onto the graphene surface. Functionalization may be achieved through π–π stacking interactions using pyrene-based linkers (Domingues et al., 2024), or through covalent attachment strategies (Dugas et al., 2004), enabling stable and specific capture of complementary malaria DNA sequences in the sensing medium.

This layered structure forms the active SPR sensing interface. Upon exposure to malaria-infected samples, changes in the refractive index near the surface result in measurable shifts in the resonance angle, enabling stage-specific detection of the infection.

Additionally, while the proposed sensor configurations demonstrate strong performance under equilibrium conditions, this study does not include an analysis of the dynamic response following target molecule introduction. Modeling such time-dependent behavior would require a kinetic framework incorporating association and dissociation rate constants, molecular diffusion coefficients, and fluidic transport conditions. These parameters are system-specific and generally derived from experimental measurements. Given the theoretical scope of this work, only steady-state optical responses were considered. Future experimental studies will be essential to evaluate the sensor’s real-time performance and to validate its applicability in time-critical diagnostic settings.

## 4 Conclusion

This study presents a theoretical design and optimization of a graphene-integrated SPR biosensor for malaria detection and stage differentiation. By combining silver, silicon nitride, graphene, and thiol-tethered ssDNA in Sys_3_ and Sys_4_ configurations, enhanced plasmonic performance was achieved. Sys_3_ demonstrated the highest sensitivity (353.14°/RIU for the ring stage), sharper resonance dips, and superior quality factor, while Sys_4_ offered improved biorecognition due to its functional layer. Both systems successfully distinguished among malaria stages based on refractive index changes, offering potential for early diagnosis and treatment monitoring. For clinical translation, challenges such as fabrication reproducibility, interfacial stability, and sample handling must be addressed. Future work should include experimental validation, assessment with real biological samples, and integration with microfluidic systems to support point-of-care deployment in endemic regions.

## Data Availability

The original contributions presented in the study are included in the article/Supplementary Material, further inquiries can be directed to the corresponding authors.
