# Supplementary material for: Design and simulation of a graphene-integrated SPR biosensor for malaria detection
Source: Front Bioeng Biotechnol. 2025 Jun 23;13:1580344. doi: 10.3389/fbioe.2025.1580344 (PMC12264435; doi:10.3389/fbioe.2025.1580344)
Supplement: Supplementary file 1 [file Table1.docx]

**Design and Simulation of a Graphene-Integrated SPR Biosensor for Malaria Detection**

**Talia Tene^1,^*, Fabian Arias Arias^2^, Karina I. Paredes-Páliz^3^, Juan Carlos González García^4^, Nataly Bonilla García^4^, Cristian Vacacela Gomez^5,^***

^1^Department of Chemistry, Universidad Técnica Particular de Loja, Loja 110160, Ecuador

^2^Department of Chemistry and Chemical Technologies, University of Calabria, Via P. Bucci, Cubo 15D, 87036 Arcavacata di Rende, Italy.

^3^Grupo de Investigación en Salud Pública, Facultad de Ciencias de la Salud, Universidad Nacional de Chimborazo, Riobamba 060108, Ecuador

^4^Facultad de Ciencias, Escuela Superior Politécnica de Chimborazo (ESPOCH), Riobamba, 060155, Ecuador

^5^INFN-Laboratori Nazionali di Frascati, Via E. Fermi 54, 00044 Frascati, Italy

Correspondence: [tbtene@utpl.edu.ec](mailto:tbtene@utpl.edu.ec) (T.T.) & [cristianisaac.vacacelagomez@utpl.edu.ec](mailto:cristianisaac.vacacelagomez@utpl.edu.ec) (C.V.G.)

**Theoretical Framework**

1. **Numerical approach**

The reflective intensity of the proposed *N^th^*-layer sensor model is calculated using the TMM [28-30]. Then, the analysis of the sensor considers boundary conditions for the tangential component, with initial limit Z = Z_1_ = 0, and final limit Z_n-1_, giving the following expression:

| $\left[ \begin{matrix} E_{1} \\ H_{1} \end{matrix} \right]=M_{ij}\left[ \begin{matrix} E_{N-1} \\ H_{N-1} \end{matrix} \right]$ | (1) |
| --- | --- |

In Equation (1), *E_1_*, *E_N-1_*, *V_1_*, and *V_N-1_* represents the tangential components of the electric and magnetic fields for the initial and *N^th^* layer, respectively. M*_ij_* indicates the transfer matrix characteristics of the *N^th^* layer model. The transfer matrix can be computed as:

| $M_{ij}=\left[ \prod_{k=2}^{N-1} M_{k} \right]_{ij}=\left[ \begin{matrix} M_{11} & M_{12} \\ M_{21} & M_{22} \end{matrix} \right]$ | (2) |
| --- | --- |

With

| $M_{k}=\left[ \begin{matrix} \cos\beta_{k} & (-i sin \beta_{k})/q_{k} \\ -i q_{k}\sin\beta_{k} & \cos\beta_{k} \end{matrix} \right]$ | (3) |
| --- | --- |

Denoting

| $\beta_{k}=\frac{2\pi d_{k}}{\lambda_{0}}\sqrt{\varepsilon_{k}-n_{1}^{2}\sin^{2} \theta}$ | (4) |
| --- | --- |

And

| $q_{k}=\frac{\sqrt{\varepsilon_{k}-n_{1}^{2}\sin^{2} \theta}}{\varepsilon_{k}}$ | (5) |
| --- | --- |

in Equation (3)-(5),$\lambda_{0}$ represents the wavelength of the incident light, $n_{1}$ is the refractive index, $\varepsilon_{k}$ represents the dielectric constant, $\beta_{k}$ represents the phase constant, $\theta$ represents the entrance angle, and $d_{k}$ represents the depth of the $k^{th}$ layer. For comparison with experiments, we adopt the use of He-Ne laser with $\lambda_{0}=633$ nm.

After straightforward computations, the total reflection of the *N^th^*-layer model can be expressed as:

| $R=\left\vert\frac{\left( M_{11}+M_{12} q_{N} \right)q_{1}-\left( M_{21}+M_{22} q_{N} \right)}{\left( M_{11}+M_{12} q_{N} \right)q_{1}+\left( M_{21}+M_{22} q_{N} \right)} \right\vert^{2}$ | (6) |
| --- | --- |

By using Equation (6), the reflectance as a function of the angle of incidence (SPR curve) can be calculated.

1. **Performance Metrics Equations**

We now move on the main performance metric of the proposed sensors. The first parameter is the sensitivity enhancement regarding the baseline sensors after/before pathogen adsorption, denoted as:

| $\Delta S_{RI}^{after}=\frac{(S_{RI}^{after}-S_{RI}^{before})}{S_{RI}^{before}}$ | (7) |
| --- | --- |

Then, the sensitivity to the refractive index change can be expressed as:

| $S_{RI}=\frac{\Delta\theta}{\Delta n}$ | (8) |
| --- | --- |

Here, $\Delta\theta$ represents the angle shift variation and $\Delta n$ represents the refractive index variation.

The detection accuracy (DA) can be expressed as in terms of $\Delta\theta$ and the full width at half maximum (FWHM) of the SPR curve, as:

| $DA=\frac{\Delta\theta}{FWHM}$ | (9) |
| --- | --- |

The Quality Factor (QF) can be expressed in terms of $S_{RI}$ and FWHM, as follows:

| $QF=\frac{S_{RI}}{FWHM}$ | (10) |
| --- | --- |

The Figure of Merit (FoM) can be expressed as:

| $FoM=\frac{S_{RI}(1-R_{min})}{FWHM}$ | (11) |
| --- | --- |

Here, $R_{min}$ represents the lowest normalized reflection value of the SPR curve.

The Limit of Detection (LoD) can be calculated as:

| $LoD=\frac{\Delta n}{\Delta\theta}\times0.005^{\circ}$ | (12) |
| --- | --- |

Finally, the Comprehensive Sensitivity Factor (CSF) ratio can be calculated:

| $CSF=\frac{S_{RI}\times(R_{max}-R_{min})}{FWHM}$ | (13) |
| --- | --- |

$R_{min}$ represents the maximum reflectance before resonance, typically at non-resonant wavelengths or angles. All computations in this investigation are done with a data sampling of $5\times{10}^{4}$ points, assuming a TM-polarized light, as required for surface plasmon excitation at the metal–dielectric interface.

**Supplementary Tables**

**Table S1.** Summary of numerical performance metrics of SPR Peak Position, Attenuation (%), Full Width at Half Maximum (FWHM), and Sensitivity Enhancement (%) for Systems Sys_0_ through Sys_4_.

| **Sys No.** | **Code** | **SPR Peak position** | **Attenuation (%)** | **FWHM** | **Enhancement (%)** |
| --- | --- | --- | --- | --- | --- |
| 0 | Sys_0_ | 68.651 | 0.019 | 0.937 | 0.0 |
| 1 | Sys_1_ | 78.162 | 0.551 | 1.611 | 13.854 |
| 2 | Sys_2_ | 84.201 | 10.336 | 2.792 | 22.650 |
| 3 | Sys_3_ | 85.335 | 35.450 | 4.157 | 24.302 |
| 4 | Sys_4_ | 86.227 | 45.373 | 4.557 | 25.602 |

**Table S2.** Summary of numerical performance metrics of SPR Peak Position, Attenuation (%), Full Width at Half Maximum (FWHM), and Sensitivity Enhancement (%) by changing the silver thickness for Sys_3_ and Sys_4_.

| **Thickness (nm)** | **SPR Peak position** | **Attenuation (%)** | **FWHM** | **Enhancement (%)** |
| --- | --- | --- | --- | --- |
| **Sys_3_** | | | | |
| 40 | 83.393 | 3.529 | 4.798 | 16.364 |
| 45 | 84.216 | 1.179 | 4.387 | 17.511 |
| 50 | 84.865 | 14.604 | 4.196 | 18.417 |
| 55 | 85.335 | 35.450 | 4.157 | 19.072 |
| 60 | 85.652 | 55.138 | 4.235 | 19.515 |
| 65 | 85.853 | 70.108 | 4.443 | 19.796 |
| **Sys_4_** | | | | |
| 40 | 84.076 | 2.090 | 4.910 | 16.465 |
| 45 | 85.020 | 3.100 | 4.580 | 17.772 |
| 50 | 85.747 | 21.505 | 4.484 | 18.780 |
| 55 | 86.227 | 45.373 | 4.557 | 19.445 |
| 60 | 86.496 | 64.896 | 4.797 | 19.817 |
| 65 | 86.628 | 77.965 | 5.315 | 20.001 |

**Table S3.** Summary of numerical performance metrics of SPR Peak Position, Attenuation (%), Full Width at Half Maximum (FWHM), and Sensitivity Enhancement (%) by changing the silicon nitride thickness for Sys_3_ and Sys_4_.

| **Thickness (nm)** | **SPR Peak position** | **Attenuation (%)** | **FWHM** | **Enhancement (%)** |
| --- | --- | --- | --- | --- |
| **Sys_3_** | | | | |
| 5 | 84.216 | 1.179 | 4.387 | 17.597 |
| 6 | 86.203 | 10.003 | 4.925 | 20.373 |
| 7 | 87.287 | 49.918 | 5.900 | 21.886 |
| 8 | 86.648 | 79.663 | 8.097 | 20.994 |
| 9 | 85.907 | 89.417 | 13.534 | 19.959 |
| 10 | 85.300 | 92.817 | 23.333 | 9.235 |
| **Sys_4_** | | | | |
| 5 | 84.076 | 2.090 | 4.910 | 16.651 |
| 6 | 85.976 | 0.128 | 5.259 | 19.287 |
| 7 | 87.561 | 22.780 | 5.812 | 21.486 |
| 8 | 87.052 | 66.056 | 7.111 | 20.780 |
| 9 | 86.305 | 82.982 | 9.460 | 19.743 |
| 10 | 85.648 | 89.717 | 15.563 | 18.832 |

**Table S4.** Summary of numerical performance metrics of SPR Peak Position, Attenuation (%), Full Width at Half Maximum (FWHM), and Sensitivity Enhancement (%) by increasing the number of graphene layers in Sys_3_ and Sys_4_.

| **No. Layers** | **SPR Peak position** | **Attenuation (%)** | **FWHM** | **Enhancement (%)** |
| --- | --- | --- | --- | --- |
| **Sys_3_** | | | | |
| L1 | 84.216 | 1.179 | 4.387 | 17.597 |
| L2 | 85.181 | 12.821 | 5.364 | 18.945 |
| L3 | 85.811 | 31.219 | 6.291 | 19.825 |
| L4 | 85.896 | 48.771 | 7.178 | 19.943 |
| L5 | 85.667 | 61.215 | 8.043 | 19.624 |
| L6 | 85.341 | 69.306 | 8.887 | 19.168 |
| **Sys_4_** | | | | |
| L1 | 85.976 | 0.128 | 5.259 | 18.198 |
| L2 | 86.683 | 13.618 | 6.183 | 19.171 |
| L3 | 86.596 | 35.252 | 7.047 | 19.051 |
| L4 | 86.207 | 50.966 | 7.872 | 18.516 |
| L5 | 85.787 | 60.907 | 8.639 | 17.939 |
| L6 | 85.392 | 67.423 | 9.345 | 17.396 |

**Table S5.** Summary of numerical performance metrics of SPR Peak Position, Attenuation (%), Full Width at Half Maximum (FWHM), and Sensitivity Enhancement (%) by changing the ssDNA layer thickness for Sys_4_.

| **Thickness (nm)** | **SPR Peak position** | **Attenuation (%)** | **FWHM** | **Enhancement (%)** |
| --- | --- | --- | --- | --- |
| **Sys_4_** | | | | |
| 3.2 | 86.683 | 13.618 | 6.281 | 18.502 |
| 5 | 86.916 | 20.447 | 6.426 | 18.820 |
| 10 | 87.027 | 43.712 | 7.005 | 18.972 |
| 20 | 86.406 | 73.623 | 9.437 | 18.123 |
| 30 | 85.776 | 84.894 | 3.766 | 17.262 |
| 50 | 85.522 | 88.928 | 1.346 | 17.975 |

**Table S6.** Summary of optimized parameters for Sys_3_ and Sys_4_. Additionally, the refractive index values for different Malaria stage have been reported.

| **Material** | **Refractive Index (RI)** | **Thickness (nm)** |
| --- | --- | --- |
| **Opt-Sys_3_** | | |
| BK7 (P) | 1.5151 | --- |
| Ag | 0.056253 + 4.2760 i | 45.0 |
| Si_3_N_4_ (SiN) | 2.0394 | 5.0 |
| Graphene | 3.0 + 1.1491 i | 0.34*L (L=2) |
| **Opt-Sys_4_** | | |
| BK7 (P) | 1.5151 | --- |
| Ag | 0.056253 + 4.2760 i | 40.0 |
| Si_3_N_4_ (SiN) | 2.0394 | 6.0 |
| Graphene | 3.0 + 1.1491 i | 0.34*L (L=2) |
| ssDNA | 1.462 | 5.0 |
| **Malaria Stage** | | |
| Ring (II) | 1.395 | --- |
| Trophozoite (III) | 1.381 | --- |
| Schizont (IV) | 1.371 | --- |

**Table S7.** Summary of numerical performance metrics of SPR Peak Position, Attenuation (%), Full Width at Half Maximum (FWHM), and Sensitivity Enhancement (%) at different Malaria stages for optimized Sys_3_ and Sys_4_.

| **Thickness (nm)** | **SPR Peak position** | **Attenuation (%)** | **FWHM** | **Enhancement (%)** |
| --- | --- | --- | --- | --- |
| **Opt-Sys_3_** | | | | |
| Normal (I) | 85.181 | 12.821 | 5.410 | 0.0 |
| Ring (II) | 82.709 | 3.214 | 4.871 | 2.902 |
| Trophozoite (III) | 79.067 | 0.157 | 4.225 | 7.177 |
| Schizont (IV) | 77.020 | 0.002 | 3.902 | 9.580 |
| **Opt-Sys_4_** | | | | |
| Normal (I) | 86.917 | 20.447 | 6.427 | 0.0 |
| Ring (II) | 84.707 | 0.753 | 5.889 | 2.542 |
| Trophozoite (III) | 80.726 | 1.864 | 5.369 | 7.122 |
| Schizont (IV) | 78.589 | 3.858 | 5.107 | 9.581 |
